# Supplementary material for: GSKIP-Mediated Anchoring Increases Phosphorylation of Tau by PKA but Not by GSK3beta via cAMP/PKA/GSKIP/GSK3/Tau Axis Signaling in Cerebrospinal Fluid and iPS Cells in Alzheimer Disease
Source: J Clin Med. 2019 Oct 21;8(10):1751. doi: 10.3390/jcm8101751 (PMC6832502; doi:10.3390/jcm8101751)
Supplement: Supplementary file 1 [file jcm-08-01751-s001.zip › Manuscript supplementary figure/supplementary figure/(JCM-R2)Supplementary figure legends.pdf]

## Supplementary figure legends

**Supplementary Figure 1. GSKIP through PKA and GSK3β mediated Tau phosphorylation as an AKAP.** SH-SY5Y cells were followed to treat with 25 μM FSK for 1 h; 40 μg of cell lysate per lane was analyzed for indicated antibodies, including anti-phosphor-Tau, and total Tau (1/1000 dilution). The results indicate that phosphorylation levels of Ser214, Ser262, and Ser409 increased after FSK treatment but decreased on Tau Ser396. The phosphorylation of Tau at Ser231 and Ser205 may not be involved in the GSKIP-mediated pathway. However, Tau Ser214, Ser409, and Ser262 were phosphorylated by PKA.

**Supplementary Figure 2. Characterization of the iPSC lines and derived neurons.** (A) Silencing of exogenous reprogramming factors and SeV vector and expression of endogenous pluripotency markers, NANOG, cMYC, KIF4, OCT4 and SOX2, were confirmed by RT-PCR. hiPSC at passage 6 was used as a positive control of SeV vector. (B) Parental AD-iPSC (*APP*$^{WT/D678H}$) and CRISPR/Cas9-edited isogenic lines (APP$^{WT/WT}$ and APP$^{D678H/D678H}$) show embryonic stem cell-like morphology (Phase in the left panels) and expressed pluripotent stem cell marker OCT4 (red in the middle panels). Scale bar, 100 μm. G-banding karyotypes of three iPSC lines are shown in the right panels. Twenty individual chromosomal spreads were analyzed for each iPSC line at passages 20-25. Of 20 metaphases, all were normal (46, XX). (C) Teratoma assay shows derivatives of all three germ layers. (D) RT-PCR analyses of gene expression in different stages: iPSC, embryoid body at day 12 (EB_D12), and induced neuron at day 9 (iN_D9). The results show expression of stem cell markers, NANOG, OCT4 and SOX2, in iPSCs, and endoderm (AFP and GATA4), mesoderm (T and RUNX1) and ectoderm (NCAM and NESTIN) markers in differentiated EBs. ACTB was used for the internal standard. (E) Design and flow diagram of neuronal differentiation from iPSCs. The method is modified from "Rapid Single-Step Induction of Functional Neurons from Human Pluripotent Stem Cells. Neuron 78, 785-798 (2013)". (F) Expression of dendritic and axonal markers, MAP2 and Smi312, respectively, in neurons derived from isogenic iPSC lines on day 29 after differentiation. Scale bar, 100 μm.

**Supplementary Figure 3. Increased Tau phosphorylation in iPSC-derived neurons from AD patients.** Sample:10 μL (4 ug) in two other groups of AD patients (*Apo*E and *PSEN*1) [41,42].
