# Supplementary figures and images for: GSKIP-Mediated Anchoring Increases Phosphorylation of Tau by PKA but Not by GSK3beta via cAMP/PKA/GSKIP/GSK3/Tau Axis Signaling in Cerebrospinal Fluid and iPS Cells in Alzheimer Disease
Source: J Clin Med. 2019 Oct 21;8(10):1751. doi: 10.3390/jcm8101751 (PMC6832502; doi:10.3390/jcm8101751)

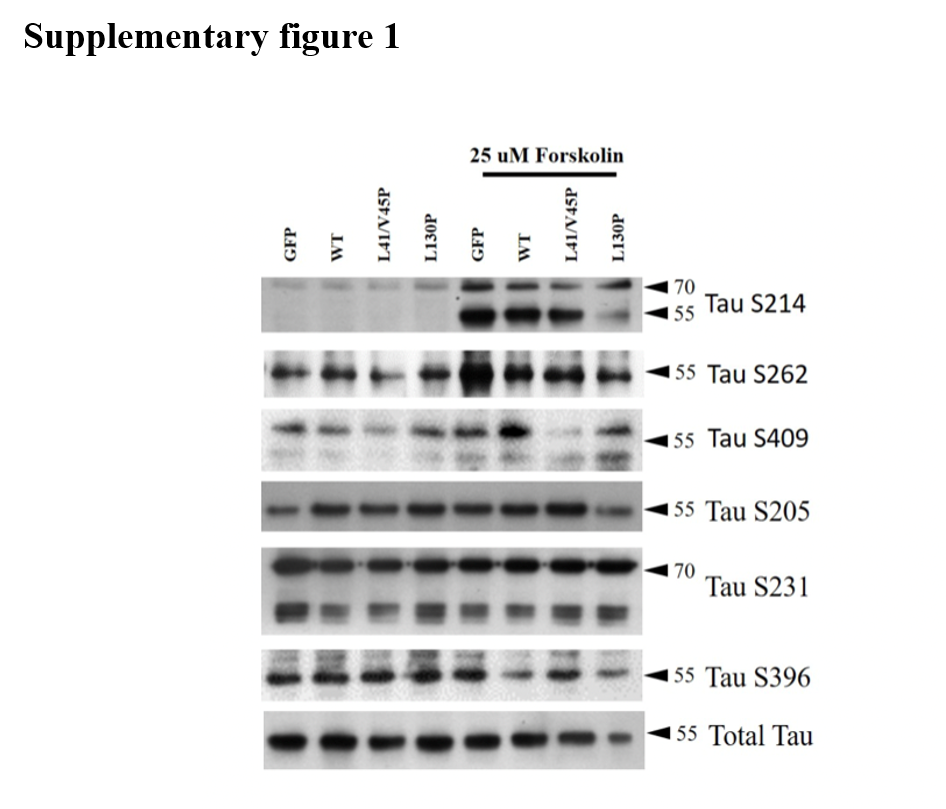

Supplement: Supplementary file 1 [file jcm-08-01751-s001.zip › Manuscript supplementary figure/supplementary figure/Supplementary figure 1.tif]

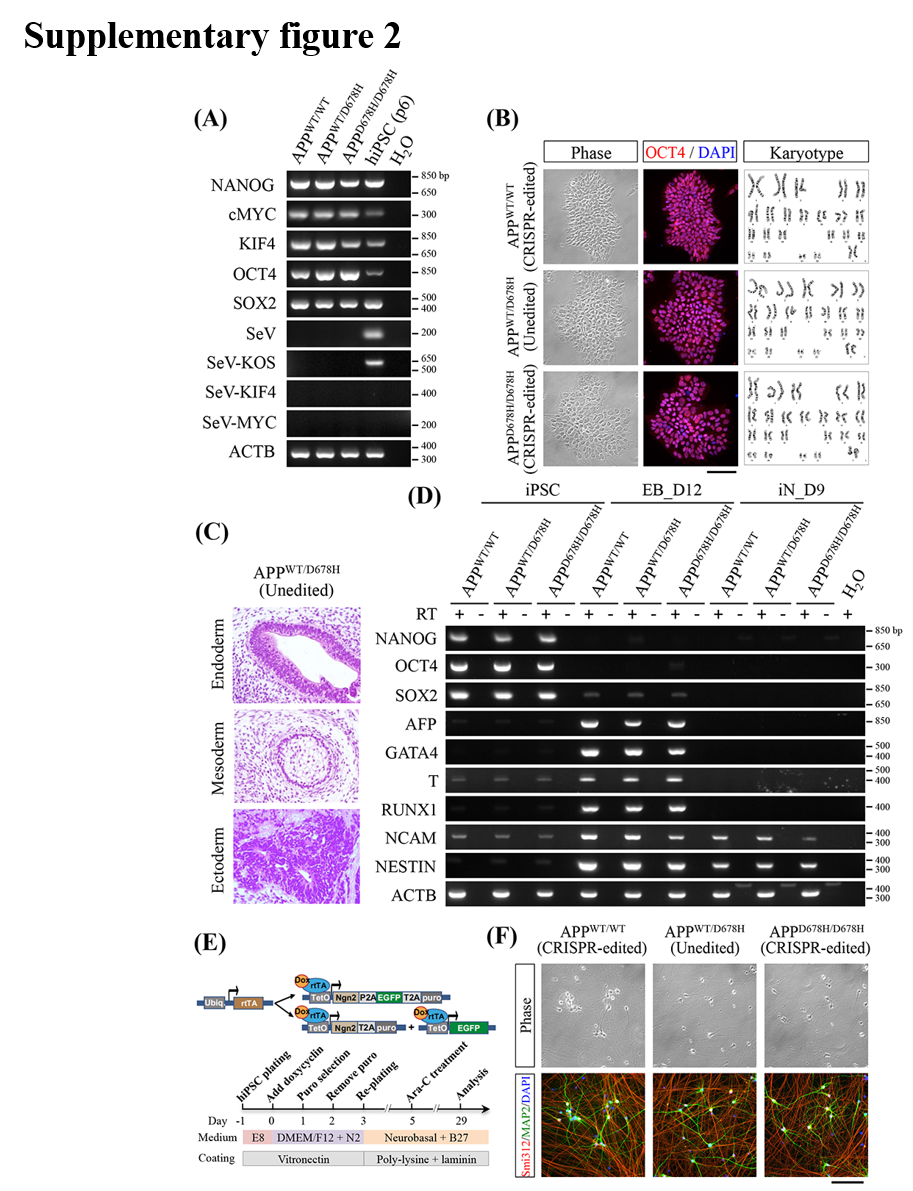

Supplement: Supplementary file 1 [file jcm-08-01751-s001.zip › Manuscript supplementary figure/supplementary figure/Supplementary Figure 2.tif]

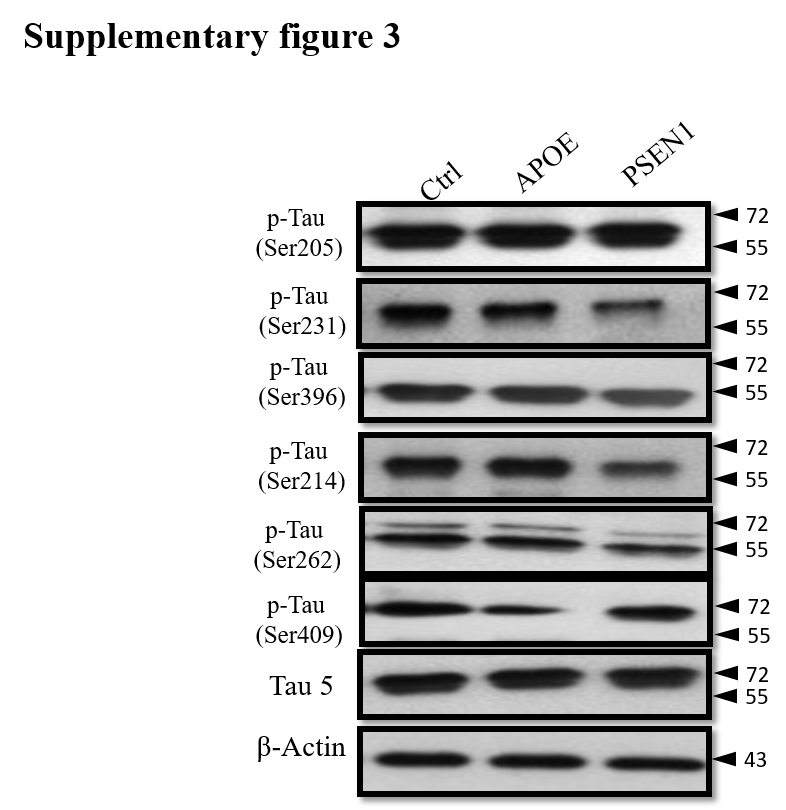

Supplement: Supplementary file 1 [file jcm-08-01751-s001.zip › Manuscript supplementary figure/supplementary figure/Supplementary figure 3.tif]

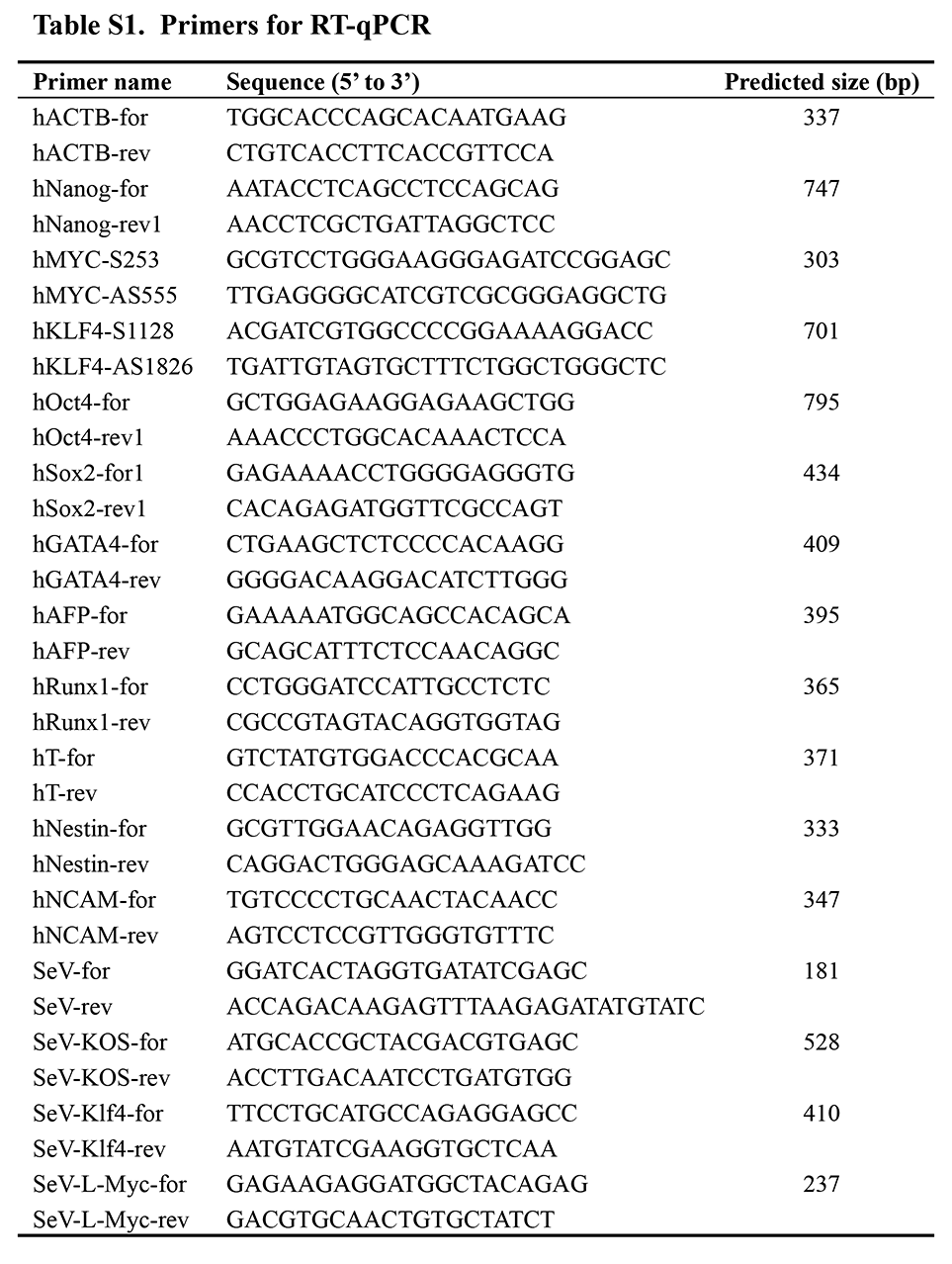

Supplement: Supplementary file 1 [file jcm-08-01751-s001.zip › Manuscript supplementary figure/supplementary table/(JCM-R2)Supplementary Table 1.tif]
